# Microwave assisted synthesis and characterisation of a zinc oxide/tobacco mosaic virus hybrid material. An active hybrid semiconductor in a field-effect transistor device

**DOI:** 10.3762/bjnano.6.81

**Published:** 2015-03-20

**Authors:** Shawn Sanctis, Rudolf C Hoffmann, Sabine Eiben, Jörg J Schneider

**Affiliations:** 1 Fachbereich Chemie, Eduard-Zintl-Institut, Fachgebiet Anorganische Chemie, Technische Universität Darmstadt, Alarich-Weiss Straße 12, 64287 Darmstadt, Germany; 2 Institute of Biomaterials and Biomolecular Systems, Dept. of Molecular Biology and Plant Virology, University of Stuttgart, 70550 Stuttgart, Germany

**Keywords:** field-effect transistor, microwave synthesis, molecular precursor, thin film transistor, tobacco mosaic virus, zinc oxide

## Abstract

Tobacco mosaic virus (TMV) has been employed as a robust functional template for the fabrication of a TMV/zinc oxide field effect transistor (FET). A microwave based approach, under mild conditions was employed to synthesize stable zinc oxide (ZnO) nanoparticles, employing a molecular precursor. Insightful studies of the decomposition of the precursor were done using NMR spectroscopy and material characterization of the hybrid material derived from the decomposition was achieved using dynamic light scattering (DLS), transmission electron microscopy (TEM), grazing incidence X-ray diffractometry (GI-XRD) and atomic force microscopy (AFM). TEM and DLS data confirm the formation of crystalline ZnO nanoparticles tethered on top of the virus template. GI-XRD investigations exhibit an orientated nature of the deposited ZnO film along the c-axis. FET devices fabricated using the zinc oxide mineralized virus template material demonstrates an operational transistor performance which was achieved without any high-temperature post-processing steps. Moreover, a further improvement in FET performance was observed by adjusting an optimal layer thickness of the deposited ZnO on top of the TMV. Such a bio-inorganic nanocomposite semiconductor material accessible using a mild and straightforward microwave processing technique could open up new future avenues within the field of bio-electronics.

## Introduction

In recent years, the synthesis and fabrication of bio-inorganic nanostructures have gained tremendous importance for the fabrication of nanoscale devices with defined functional properties [1–3]. Significant interest has been dedicated to the generation of multifunctional devices by employing a unique combination of functional biological molecules and inorganic materials. The use of biological building blocks at the nanoscale include DNA, peptides, bacteriophages and viruses which exhibit diverse properties for the controlled formation of devices with possible application in areas such as sensors, photonics, energy storage as well as electronic transistors [4–8]. Fabrication of necessary functional hybrid materials often require well-defined 1D and 2D biological molecules as structure-directing agents, enabling a "bottom-up" approach for building these complex nanoarchitectures. Among the several biological templates, the tobacco mosaic virus (TMV) has shown great potential to function as a robust biological template for the deposition of a variety of inorganic materials under mild fabrication conditions. With its well-defined tube-like structure, the tobacco mosaic virus is one the most widely studied plant virus consisting of ≈2130 identical protein units, a length of 300 nm and an outer and inner diameter of 18 nm and 4 nm, respectively. It is also displays a remarkable stability for temperatures of up to about 60 °C in a pH range between 2 and 10. Such a rigid nanostructured template offers an interesting opportunity for the directed assembly of inorganic, metallic and semiconducting materials [9–10].

With the aim to generate defined semiconducting nanostructures in the nanometer range, deposition of ZnO nanoparticles onto the wild type TMV (wt TMV) presents itself to be an ideal choice of material combination.

Zinc oxide (ZnO) is one the most widely studied, non-toxic, n-type semiconducting inorganic oxide with a direct band-gap of ≈3.37 eV. This enables the fabrication of functional zinc oxide based transistors [11–12]. The ability to fabricate zinc oxide based transistors from various precursor solutions, under mild basic conditions, makes it a suitable candidate to be deposited upon the TMV template [13]. We have previously reported on the synthesis of air-stable, Schiff base type, molecular zinc complex diaqua-bis[2-(methoxyimino)propanato]zinc(II) which represents an ideal molecular single source precursors for the fabrication of functional zinc oxide transistors at low-temperatures [14]. Employment of such a class of molecular precursor complexes, with a low decomposition temperature and volatile and well defined byproducts ensures the formation of a resultant zinc oxide material with high purity. Additionally, microwave assisted decomposition of this class of precursors in solution has shown to yield stable colloidal nanoparticle dispersions [15–16]. In order to assist the in situ deposition of nanoparticulate zinc oxide onto the wt TMV template, mild microwave synthesis conditions for the zinc oximate precursor were used by us for the first time.

Herein, we report on the fabrication of a functional hybrid semiconducting material based on a microwave assisted ZnO mineralization of the TMV [17]. The resultant TMV/ZnO nanoscale hybrid material exhibits functional transistor behaviour with a reasonable performance without any post-processing at higher temperature.

## Experimental section

All reagents were purchased from Sigma-Aldrich or Carl Roth and used as received unless otherwise stated. The molecular precursor, diaqua-bis[2-(methoxyimino)propanato]zinc(II), referred to as the zinc oximato complex in this work, was synthesized as previously reported [14]. The deposition solution was prepared by mixing solutions of the zinc oximato complex and polyvinylpyrrolidone (PVP) (mol. wt ≈ 10k) in methanol and drop wise addition of a solution of tetraethylammonium hydroxide (TEAOH) in methanol, so that the final concentrations were [Zn^2+^] = 10 mM, [PVP] = 10 mM, and [TEAOH] = 12.5 mM. The microwave reactions were performed in a Discover (CEM Corporation) microwave oven using commercially available glassware supplied by the manufacturer. A few drops of 0.5 mM aqueous zinc acetate solution were deposited on the FET substrate for 5 min and excess was removed and blow-dried under a stream of argon flow. Thereafter, a drop TMV (0.5 μL, 0.5 mg/mL) was placed onto the FET substrate surface and incubated for 10 min. The excess virus suspension was removed by blow drying the substrate under a mild argon flow. The positively charged zinc cations facilitate the efficient immobilization of the negatively charged TMV particles on to the substrate. The FET substrate with the immobilized TMV was immersed in the microwave reaction vessel containing 10 mL of the reaction solution. Reactions were carried out by heating for 30 min, with a maximum applied power of 50 W (dynamic power mode), with an average power of ≈15 W, throughout the experiment. The prefabricated FET substrates (Fraunhofer IWS, Dresden) were sequentially cleaned in an ultra-sonic bath with acetone, DI-water and iso-propanol, respectively for ten minutes each, prior to its immersion in the microwave vessel containing the reaction solution. Substrates for the FET devices (15 × 15 mm^2^) consisted of n-doped silicon with a 90 nm layer of SiO_2_, on which gold electrodes were attached with an intermediate adhesion layer of indium tin oxide. The electrodes possessed an inter-digital structure with a channel width W of 10 mm and a channel length of 20 µm.

^13^C nuclear magnetic resonance spectroscopy (NMR) was undertaken using a DRX500 (Bruker) spectrometer. Experiments to study the decomposition of the precursor in the microwave were performed by preparing the reaction solution (with and without the TEAOH) in tetra-deuteromethanol (methanol-*d*_4_). The reactions were performed in the absence of the virus to avoid any influence from the TMV. For the NMR studies the decomposed precursor solution after microwave processing was filtered through a 0.22 µm PTFE syringe filter and was directly analyzed using NMR. Transmission electron microscopy (TEM) was performed using Tecnai F20 G20 (FEI) electron microscope working at 200 kV, using lacey carbon coated copper grids. Dynamic light scattering (DLS) measurements for the ZnO suspensions were carried out using a Zetasizer Nano (Malvern). Atomic force microscopy was performed with CP-II (Bruker-Veeco) microscope using ultra sharp silicon cantilevers. Optical profilometry measurements were performed using the optical Profilometer-NewView 6200 (Zygo). Grazing incidence XRD (GI-XRD) investigations were performed with a Seifert PTS 3003 diffractometer using a Cu anode and a graphite monochromator with an applied current and voltage of 40 mA and 40 kV, respectively. FET characterizations were measured in the dark, using an HP 4155A semiconductor parameter analyzer (Agilent) in a glove box under constant O_2_ and H_2_O (<0.5 ppm). Charge carrier mobility in the saturation regime µ_SAT_ and the threshold voltage *V*_th_ were derived from a linear fitting of the square root of the drain-source current (*I*_DS_) as a function of the gate–source voltage (*V*_GS_).

Tobacco mosaic virus strain U1 was propagated in Nicotiana tabacum ‘Samsun’ nn plants for 25 days and purified according to the modified protocol of Gooding and Hebert [18].

## Results and Discussions

### Synthesis and characterization of the wt TMV/ZnO hybrid material

In order to facilitate the controlled mineralization of zinc oxide onto the TMV template, the microwave conditions for synthesis of the zinc oxide in due consideration of the stability of the TMV template had to be optimized. Use of the molecular precursor diaqua-bis[2-(methoxyimino)propanato]zinc(II) [14,19] (referred to as – the zinc oximato complex – in the following) as a source of zinc oxide was employed, herein. Solutions for the controlled formation and deposition of the ZnO nanoparticles were obtained by using a methanolic solution of zinc complex as a zinc source and polyvinylpyrrolidone (PVP) as a growth inhibiting and stabilizing agent for the zinc oxide nanoparticles. PVP has been reported to have a higher efficiency in suppressing the growth of zinc oxide during its formation, in comparison to other polymeric additives [20]. Additionally, a defined amount of tetraethylammonium hydroxide (TEAOH) was added to the precursor solution to create a mild basic environment which assists the controlled formation of the ZnO nanoparticles.

**Figure 1:**
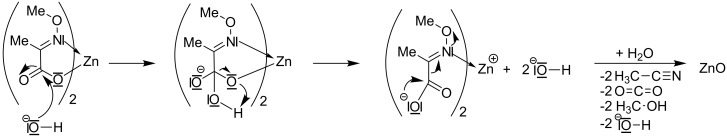
Schematic representation of the microwave decomposition pathway of the zinc oximato precursor in the presence of TEAOH as a base based on NMR spectroscopy. Note that the water ligands are omitted in the starting compound.

The addition of the optimal amount of the base TEAOH provides mild but sufficient basic conditions to ensure an efficient decomposition of the zinc complex at a low temperature as 60 °C (±3 °C) enabling the successful formation of crystalline zinc oxide. An increased reaction rate, although, with a rapid formation of zinc oxide resulting in unstable aggregates ranging up to several hundred nanometres in size, was observed in the presence of higher amounts of the base. In order to gain insight into the microwave decomposition process of the molecular precursor, the microwave decomposition process was studied in the absence and presence of the base TEAOH using ^13^C NMR spectroscopy. Without the addition of the base, the precursor complex did not undergo any decomposition after the completion of the microwave reaction under typical reaction conditions. However, in the presence of the base, the precursor does undergo decomposition with appearance of a characteristic ^13^C chemical shift corresponding to the formation of acetonitrile (δ = 117.30 ppm) under post decomposition conditions of the precursor complex. Additional chemical shifts from still coordinated as well as from residual free ligands were also present as expected due to the base-catalyzed decomposition of the precursor complex (see Supporting Information File 1, Figure S2). The products observed in the microwave initiated decomposition of the oximato complex is in full accordance with a second-order type Beckmann rearrangement reaction as observed for its solution based thermal decomposition pathway [13]. Besides the characteristic signals of the decomposition products, chemical shifts from the undecomposed precursor are still observed after the completion of the microwave reaction. Attempts to completely decompose the precursor were not pursued since long reaction time lead to the formation of undesirable precipitates of zinc oxide aggregates, in a very similar way as addition of excess base TEAOH does. Thus it can be concluded that the microwave decomposition reaction has to be fine tuned in order to obtain pure precipitates of the desired nanoscale ZnO product. A comprehensive reaction and decomposition pathway of the precursor can thus be proposed on the basis of the observed decomposition products from the described NMR experiments (Figure 1).

**Figure 2:**
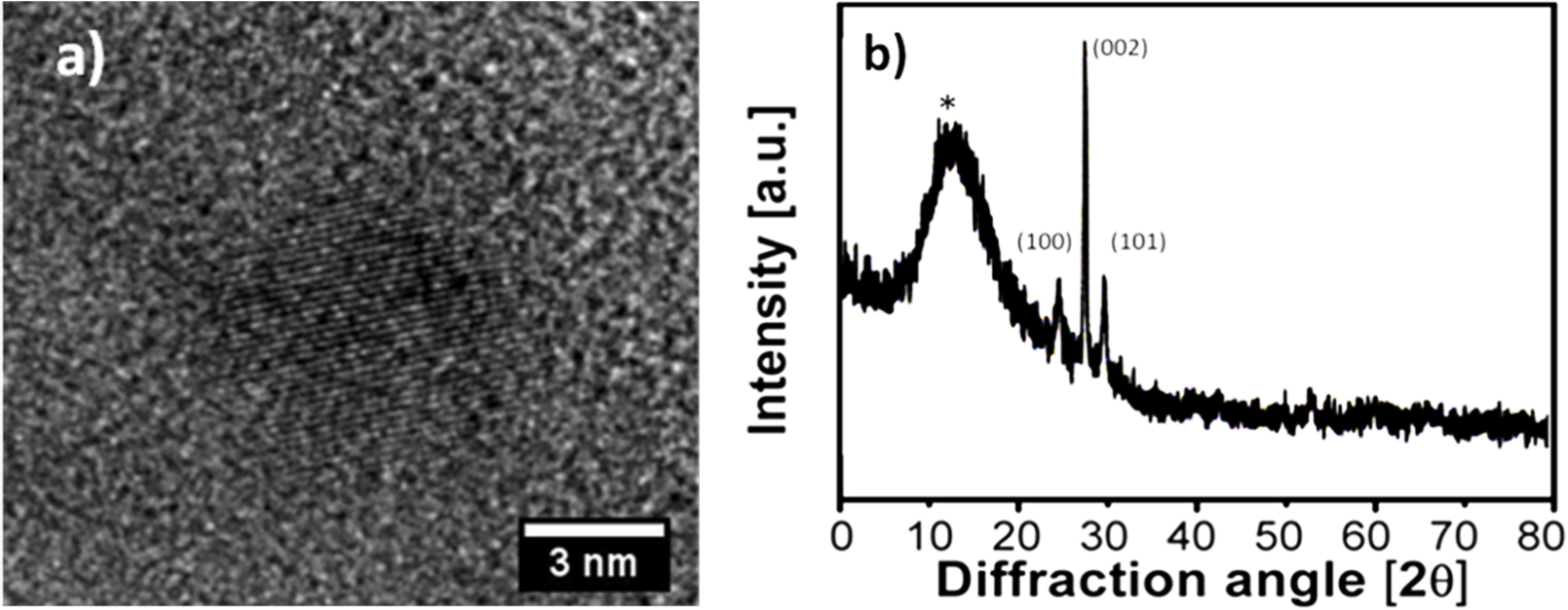
a) HRTEM image of the ZnO nanoparticle obtained from solution and b) GI-XRD spectra of the ZnO thin film after 6 deposition cycles (* = peak intensity arising from the Si/SiO_2_ substrate).

Systematic experiments have shown that a maximum microwave power of 50 W, temperature of 60 °C (±2 °C) and a synthesis time of 30 min display the optimum conditions leading to the formation of nanocrystalline ZnO. Dynamic light scattering measurements indicate the formation of stable ≈5 nm particles, after a synthesis period of 30 min (see Supporting Information File 1, Figure S1). The indicated particle size is in good agreement with HRTEM investigations of the as-synthesized particles formed from the precursor solution, which yield stable zinc oxide nanoparticles (Figure 2a). TEM also indicates the successful formation of ZnO nanocrystals in solution after the completion of the microwave irradiation process. Grazing incidence X-ray diffractometry (GI-XRD) analysis was employed to gain a deeper insight into the crystallinity of the as deposited ZnO thin film. The ZnO films display a polycrystalline nature of the ZnO being essential for the formation of an active semiconducting layer. Reflection peaks corresponding to the (100), (002) and (101) planes are characteristic of the zincite structure (Figure 2b). A greater intensity in the direction of the c-axis (i.e., perpendicular to the (002) plane) was observed. ZnO nanoparticles undergo an oriented attachment during thin film formation in the presence of PVP which hints at texturing of the ZnO nanoparticles within the deposited ZnO film [21–22].

**Figure 3:**
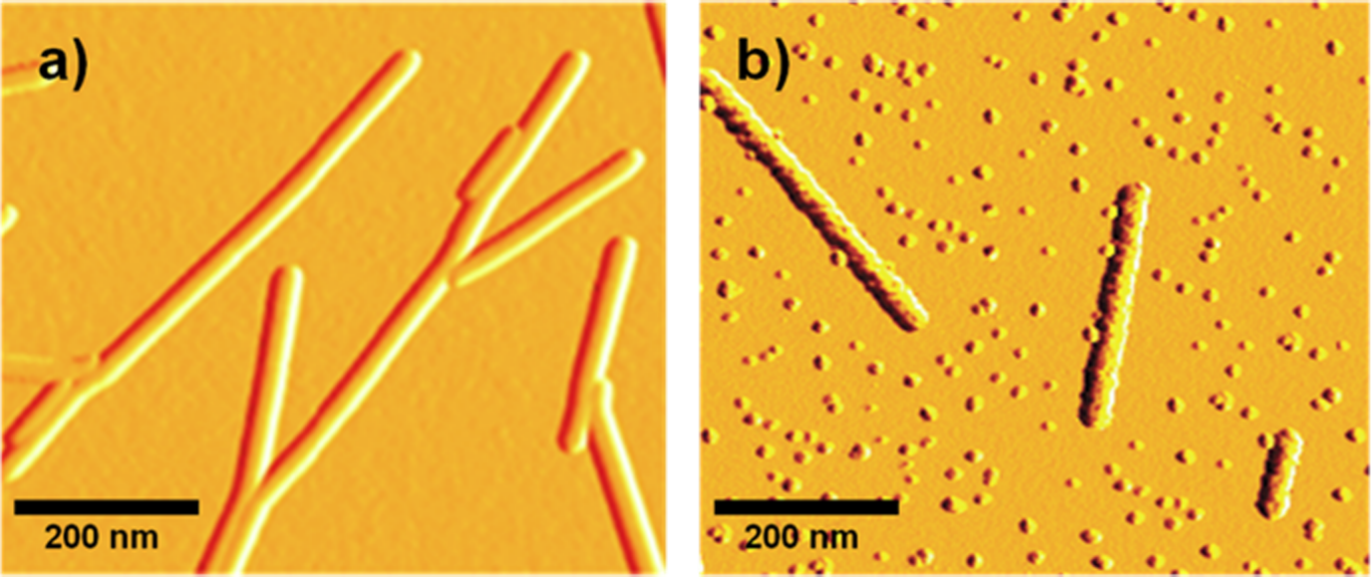
AFM micrographs of (a) the bare wt TMV template immobilized on a Si/SiO_2_ substrate as well as (b) the wt TMV template after 1 cycle of ZnO mineralization.

In order to perform an in situ microwave-based mineralization of the TMV, it is essential to ensure that the virus particle adheres to the substrate during the microwave irradiation. Intense microwave irradiations are known to have a strong tendency to denature proteins and cause potentials damage to its structural integrity [23]. Additionally, prolonged high-power microwave irradiation could lead to unexpected heating of the substrate onto which the TMV are immobilized. The substrates with the docked virus were thus immersed in a control methanolic solution containing the predetermined amounts of the TEAOH and PVP in the absence of the zinc precursor. These substrates were then subjected to the desired microwave irradiation time of 30 min. This ensures that the virus particles do not detach from the substrate. AFM investigations for a control experiment reveal an intact TMV template on the Si/SiO_2_ substrate, even after 30 min of mild microwave irradiation showing no visible deformation of its rod-like structure and its original dimensions and morphology (Figure 3a). Irradiation of the reference solution containing the virus-coated substrate with higher microwave power led to uncontrolled, rapid increase of the solution temperature and boiling of the solvent methanol (bp ≈ 65 °C). This led to a detachment of the viruses as no visible virus structures afterwards could be detected on the substrate surface by AFM analysis after this procedure. Once the reliable microwave conditions of the virus attachment and the retention of its structural integrity were confirmed, the virus-coated substrates were immersed in the reaction solution which then leads to the mineralization of the zinc oxide nanoparticles onto the virus template. Such successful mineralization of the zinc oxide onto the TMV template is obtained under low power microwave assisted decomposition of the precursor solution (Figure 3b). Hence, reliable docking of the TMV onto the silicon/silicondioxide (Si/SiO_2_) and simultaneous formation of zinc oxide nanoparticles could be achieved. AFM analysis for the bare TMV layer as well as the ZnO mineralized TMV layer after one deposition cycle reveal an average layer thickness of 15.5 nm and 25.8 nm respectively (see Supporting Information File 1, Figure S3).

**Figure 4:**
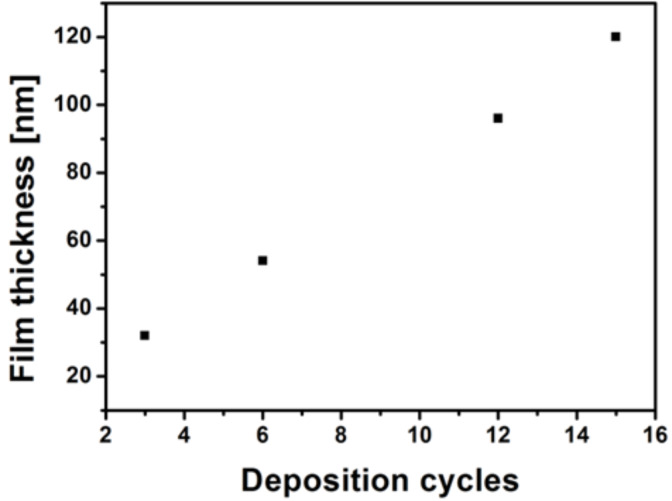
Overall thickness of the wt TMV/ZnO hybrid material as a function of the number of deposition cycles as determined by optical profilometry. ZnO was deposited onto the wt TMV template with an increasing number of deposition cycles from the precursor solution.

In order to ensure an optimum thickness and to promote a good transistor performance ZnO layers with increasing number of deposition cycles were analyzed. The increasing thickness of the ZnO films after various cycles was measured using optical profilometry (Figure 4).

The thickness and uniformity of the deposited semiconductor layer bear a crucial importance for FET device performance [12]. Variation in the layer thickness severely affects the transistor performance. For example, a thicker layer increases the resistance across the active material, while thinner layers could possibly lead to non-uniform layer deposition [24]. Therefore the layer thickness should be optimized according to these parameters.

### Field effect transistor (FET) properties

In order to assess its FET properties, the wt TMV/ZnO hybrid material template was realised in a bottom gate, bottom contact FET geometry, by employing pre-fabricated FET substrates with external gold electrodes. All fabricated devices exhibit functional transistor properties without any post-processing treatment. As a reference we had measured the electrical characteristics of microwave processed bare nanoscale ZnO without TMV, obtained again from the molecular zinc oximato precursor complex under similar conditions. These results showed only noisy and almost indiscernible signals (measurements not shown). This fact substantiates the point that the FET properties are indeed intrinsic for the microwave processed wt TMV/ZnO hybrid material. Similar results have been reported for thermally processed bare nanoscale ZnO material using the zinc oximato complex as precursor [13].

**Figure 5:**
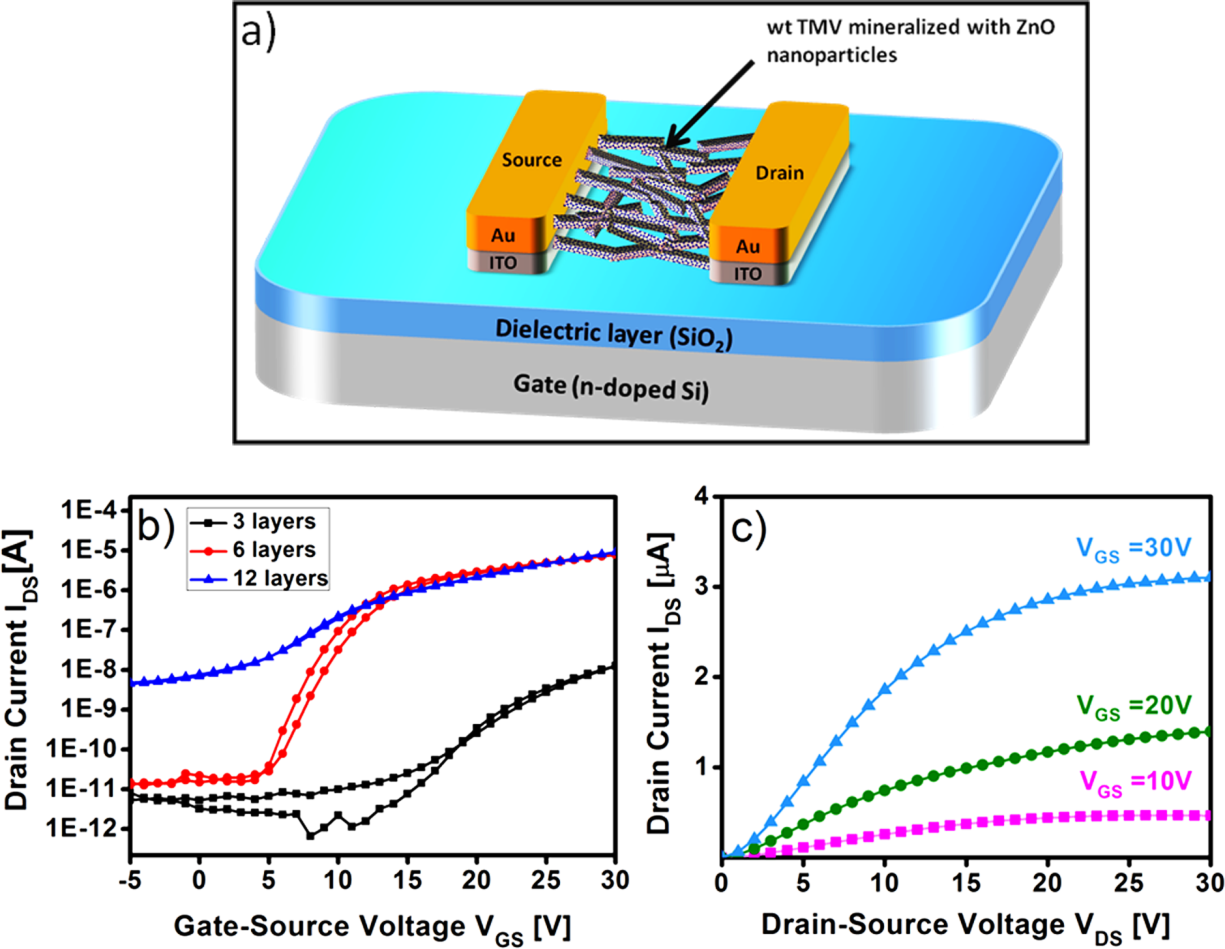
Schematic representation of the wt TMV/ZnO based FET device (a). Performance of the FET device fabricated by increasing the number of ZnO deposition cycles on the wt TMV template; (b) Transfer characteristics for constant drain–source voltage at 30 V; (c) output characteristics of TMV/ZnO hybrid after 6 deposition cycles, obtained at drain–source voltage of 30 V, for gate–source voltage varied from 0–30 V in 10 V steps.

The transistor behaviour of the wt TMV/ZnO hybrid material was then optimized based on the crucial characteristic FET values, current on/off ratio (*I*_on/off_), threshold voltage (*V*_th_) and charge carrier mobility (µ) which are considered to be essential parameters for the FET performance. Based on these performance parameters, the electrical characteristics of the devices display a stark contrast due to the difference in the number of ZnO deposition cycles which led to the formation of thicker layers. FET characteristics of the ZnO films with increased layer thickness are displayed below (Figure 5 and Table 1).

**Table 1:** Characteristic values for field-effect mobility μ, threshold voltage (*V*_th_), and on/off current ratio (*I*_on/off_) of wt TMV/ZnO hybrid material based transistor devices.

ZnO deposition cycles	µ (mobility in cm^2^/Vs)	*V*_th_ (V)	*I*_on/off_

3	8.0 × 10^−6^	17.79	10.2 × 10^2^
6	6.7 × 10^−4^	4.76	9.0 × 10^5^
12	8.4 × 10^−4^	6.80	2.1 × 10^3^
15	1.6 × 10^−3^	12.02	1.0 × 10^2^

Fewer deposition cycles (3 cycles) for the ZnO exhibited very weak transfer characteristics with significantly low On and well as Off currents, high *V*_th_ and poor mobility values. On the other hand, increased number of deposition cycles (12 cycles) of the ZnO led to a slight increase in the mobility values. Also, a positive increase in the On currents accompanied by a drastic increase in the Off currents led to a deteriorated *I*_on/off_ ratio. Moreover, an undesirable higher *V*_th_ value was observed. Further increase in the number of deposition cycles (15 cycles) deteriorated the overall transistor performance giving no significant semiconducting properties. For an optimum number (6 cycles) of ZnO deposition, the best overall FET performance values were obtained with a field-effect mobility (µ) of 6.7 × 10^−4^ cm^2^/Vs, *V*_th_ of +4.7 V and an *I*_on/off_ of 9.0 × 10^5^. A higher *I*_on/off_ in comparison to previously reported values could possibly be attributed to a greater degree of ZnO nanoparticle orientation on the wt TMV template resulting from the use of the molecular precursor complex employed [25].

## Conclusion

The ability of the virus template to maintain its structural integrity under mild microwave radiations, while facilitating the deposition of zinc oxide nanoparticles has been implemented for virus-based templating of inorganic nanomaterials, towards functional devices with diverse applications. We have successfully employed a molecular precursor to synthesize zinc oxide nanoparticles, for an in situ deposition on to a virus template. A facile, microwave-assisted approach for generating a TMV/ZnO hybrid bio-inorganic material has been implemented. We confirmed the clean in situ decomposition of the molecular precursor under mild conditions as well as the desired zinc oxide phase formation by resonance, diffractometry and microscopic methods. Moreover, the as-synthesized hybrid material has been successfully employed in a FET device. The best FET performance has been achieved by systematically controlling the thickness of the deposited zinc oxide films. The fabricated FET shows a reasonable performance for the as-prepared device, without any post processing of the bio-inorganic hybrid nanomaterial. Such an approach towards generation of a bio-inorganic material encourages the use of nanoscale virus templates to obtain hybrid materials with functional properties that can be implemented into future device applications.

## Supporting Information

File 1 Additional figures.
