# Supplementary material for: Microwave assisted synthesis and characterisation of a zinc oxide/tobacco mosaic virus hybrid material. An active hybrid semiconductor in a field-effect transistor device
Source: Beilstein J Nanotechnol. 2015 Mar 20;6:785–91. doi: 10.3762/bjnano.6.81 (PMC4419583; doi:10.3762/bjnano.6.81)
Supplement: File 1 — Additional figures. [file Beilstein_J_Nanotechnol-06-785-s001.pdf]

Supporting Information

for

Microwave assisted synthesis and characterisation of a zinc oxide/tobacco mosaic virus hybrid material. An active hybrid semiconductor in a field-effect transistor device

Shawn Sanctis[1], Rudolf C. Hoffmann[1], Sabine Eiben[2] and Jörg J. Schneider[*,§,1]

Address: [1]Fachbereich Chemie, Eduard-Zintl-Institut, Fachgebiet Anorganische Chemie, Technische Universität Darmstadt, Alarich-Weiss Straße 12, 64287 Darmstadt, Germany and [2]Institute of Biomaterials and Biomolecular Systems, Dept. of Molecular Biology and Plant Virology, University of Stuttgart, 70550 Stuttgart, Germany.

Email: Jörg J. Schneider - joerg.schneider@ac.chemie.tu-darmstadt.de

* Corresponding author

[§] Fax: +49 6151 163470; Tel: +49 6151 163225

Additional figures

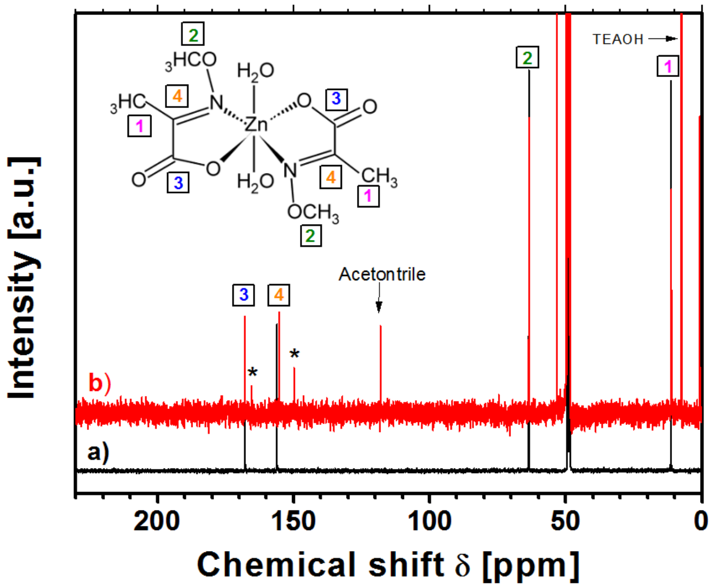

Figure S1: ^{13}C NMR spectra of reaction products after the microwave synthesis with and in the absence of the base (TEAOH). a) Zinc oximato complex without the base and b) zinc oximato complex in the presence of TEAOH as a base. Chemical shift highlighted with (*) correspond to the uncoordinated precursor ligands which arise during the microwave decomposition.

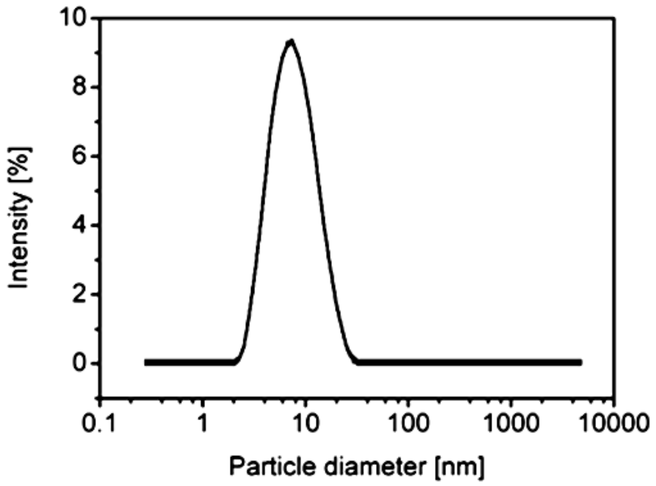

Figure S2: Particle size distribution of the formed ZnO in solution with an average diameter of ≈5 nm.

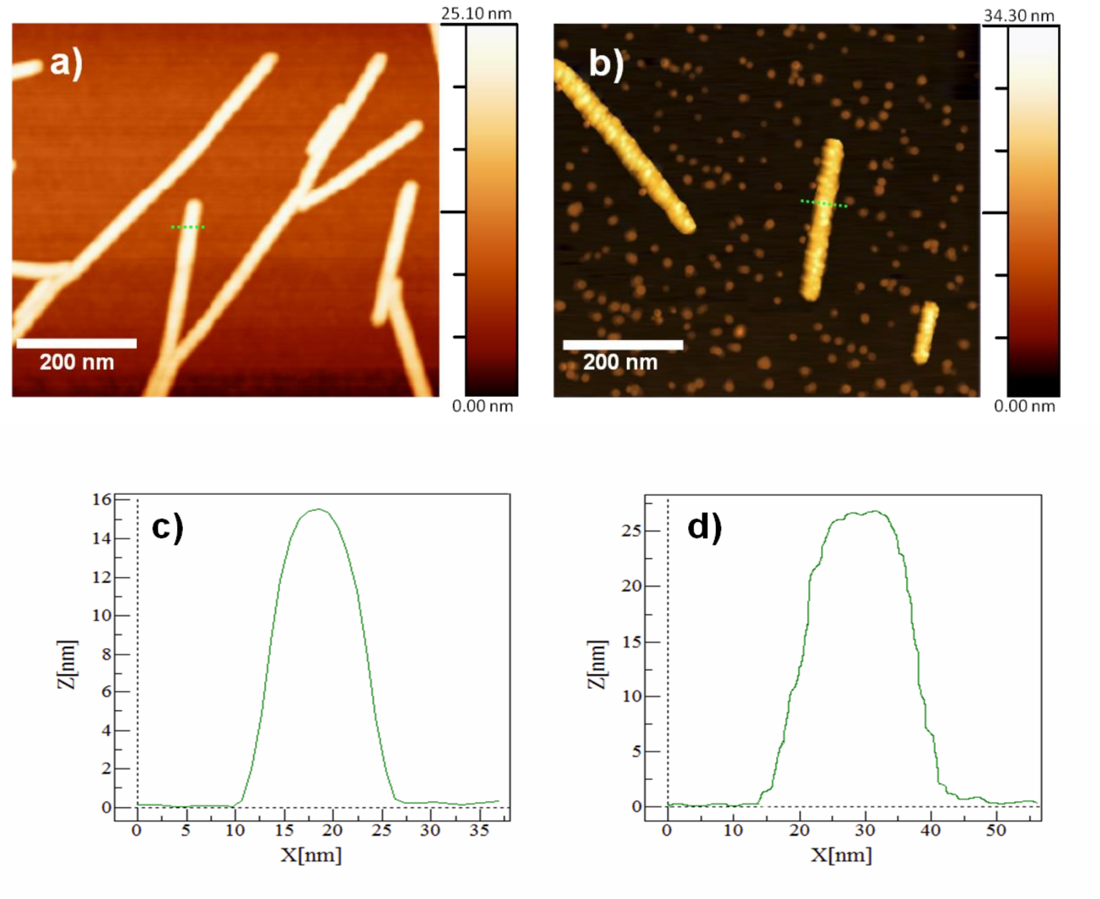

Figure S3: AFM height micrographs and of bare TMV wt template immobilized on Si/SiO_2 substrate without mineralization (a) and after 1 cycle of ZnO mineralization (b). AFM height profiles (c) and (d) measured over the region indicated with a dotted line in (a) and (b), respectively.
